# Prevalence of Irritable Bowel Syndrome Among Physicians at General Governmental Hospitals in Jeddah, Saudi Arabia

**DOI:** 10.7759/cureus.68355

**Published:** 2024-09-01

**Authors:** Raghad Rawa, Hani A Alghamdi

**Affiliations:** 1 Preventive Medicine, Saudi Board of Preventive Medicine, Jeddah, SAU; 2 Preventive Medicine Department, Public Health Directorate, Ministry of Health, Jeddah, SAU

**Keywords:** lifestyle factors, demographic factors, prevalence, physicians, irritable bowel syndrome (ibs)

## Abstract

Background

Irritable bowel syndrome (IBS) is a prevalent functional gastrointestinal disorder characterized by symptoms such as abdominal pain and altered bowel habits. It significantly impacts the quality of life and imposes a financial burden on healthcare systems. Previous studies have shown varying prevalence rates of IBS among different populations.

Objective

This study aims to determine the prevalence of IBS among physicians working in general governmental hospitals in Jeddah, Saudi Arabia, and to analyze the associated demographic and lifestyle factors.

Methods

An analytical cross-sectional study of 391 physicians from King Fahad and East Jeddah General Hospitals used an anonymous electronic survey covering demographics, health, lifestyle, and the Birmingham IBS Symptoms Questionnaire. Data were analyzed with SPSS version 29 (IBM Corp., Armonk, NY, USA) using Kruskal-Wallis and Mann-Whitney U tests, with p < 0.05 as significant.

Results

The prevalence of IBS among the participants was 45% (n=176). Significant associations were found between Birmingham scores and various demographic and lifestyle factors. Younger age groups (25-29 years, 51.4%, n=201) had higher mean ranks (212.98) compared to older age groups, with a p-value of .009. Males (54.5%, n=213) had a significantly higher mean rank (213.37) compared to females (45.5%, n=178; 175.22) (p<.001). Non-smokers (38.1%, n=149) had a significantly higher mean rank (214.27) compared to smokers (61.9%, n=242; 166.33) (p<.001). Physical exercise was associated with a lower prevalence of IBS symptoms, with non-exercisers (39.9%, n=156) having a higher mean rank (207.67) compared to exercisers (60.1%, n=235; 178.42) (p=.012). Additionally, 46.3% (n=181) of participants reported missing work due to IBS symptoms.

Conclusion

The study found a high prevalence of IBS among physicians in Jeddah, with significant associations between IBS symptoms and various demographic and lifestyle factors. These findings highlight the need for increased awareness, regular screening, and support for physicians suffering from IBS to improve their quality of life and job performance.

## Introduction

Irritable bowel syndrome (IBS) is a functional gastrointestinal disorder with symptoms like abdominal pain associated with changes in stool form or frequency [1]. IBS can lower health-related quality of life (HRQOL), reduce job efficiency, and increase healthcare costs [2]. Although IBS is not life-threatening, it can significantly impact the quality of life, affecting educational, social, and professional success [3]. IBS is often more prevalent in people with psychiatric comorbidities and young adult women than in the general population [1]. It is projected to affect 11.2% of the world's population [4]. In Asia, the prevalence of IBS is estimated to be between 5% and 10%, which is lower than in Western countries. IBS places a financial strain on the healthcare system. In the United States, the direct annual cost of diagnosing and treating IBS is projected to be between $1.7 and $10 billion. The indirect costs of absenteeism, missed workdays, and disability would double the total amount [5].

Healthcare is a significant aspect of society, with individuals involved in this field facing more stressful conditions compared to other fields. So far, only a few studies have investigated the prevalence of IBS among health professionals. A cross-sectional study among Canadian medical students reported that the prevalence of IBS was 22% [6]. The prevalence of IBS has also been reported in nurses. A study reported that more than 30% of nurses are affected by IBS. This number becomes particularly higher (48%) in nurses who work rotating shifts [7]. Although many studies have been performed in Saudi Arabia to estimate the prevalence of IBS, the prevalence of IBS has been rarely documented among physicians. IBS was found to be prevalent in 31.8% of medical students and interns in a study conducted at King Abdul-Aziz University in Jeddah [5]. According to a study in Jeddah, 31.8% of medical students and interns have IBS [8]. Another study found that among medical students in Al-Kharj, the prevalence is 21% [9].

In the present study, we aimed to assess the prevalence of IBS in physicians in Jeddah city. Due to the high-stress working environment, we hypothesized that the prevalence of IBS would be higher compared to the general population.

## Materials and methods

This study is an analytical cross-sectional study, focusing on physicians working at general governmental hospitals in Jeddah, Saudi Arabia. The inclusion criteria encompassed all physicians working at King Fahad General Hospital and East Jeddah General Hospital. Exclusion criteria included subjects with pre-existing gastrointestinal disorders such as Crohn's disease and ulcerative colitis, medical students, and non-physicians including nurses and technicians.

The primary outcome of the study was IBS prevalence and severity among physicians, measured by Birmingham IBS Symptoms Questionnaire scores. Independent variables included demographic, lifestyle, and medical history factors. The study examined associations between these variables and IBS symptoms among participating physicians.

A simple random sampling method was employed to select the study participants. The sample size was determined using the Raosoft electronic sample size calculator, based on population data from the Saudi General Authority for Statistics in Jeddah (2010). With an accepted margin of error of 5%, a confidence level of 95%, and a power of 80%, the minimum calculated sample size was set at 377. The data was ultimately collected from 391 participants to ensure a slightly larger sample, which can help account for potential dropouts or incomplete responses.

The study was conducted at King Fahad General Hospital and East Jeddah General Hospital. A comprehensive list of all working physicians at these hospitals was obtained from the human resources department. Using a simple random sampling method, participants were selected and invited to complete an anonymous electronic survey via WhatsApp, using Google Forms.

Participants completed a self-reported questionnaire, which was developed by the researchers and face-validated, administered electronically, which consisted of three sections. The first part included seven questions covering demographic information such as age, marital status, qualification, and the name of the organization. The second part comprised nine questions related to health and lifestyle factors, including previous diagnoses of IBS and lifestyle habits. The third part contained 11 questions from the Birmingham IBS Symptoms Questionnaire, with response options ranging from "All of the time" to "None of the time" [10].

Ethical approval (IRB number: A01821) was obtained from the institutional review board (IRB) of the Research and Studies Department at the Directorate of Health Affairs in Jeddah before the study began. Comprehensive informed consent was provided to all participants, emphasizing the importance of confidentiality and privacy protection throughout the study. Consent was verified through an initial question at the study's onset, ensuring that only those who agreed proceeded to complete the formal consent form. The collected data was treated with strict confidentiality and was accessible only to the principal authors for research purposes.

Statistical analysis

All statistical analyses were conducted using SPSS version 29 for MacOS (IBM Corp., Armonk, NY). Descriptive statistics were used to summarize the demographic and clinical characteristics of the participants. Categorical variables were expressed as frequencies and percentages, while continuous variables were presented as means and standard deviations.

Inferential statistics were applied to determine the associations between demographic characteristics and Birmingham scores. As the data was found to be non-normally distributed using the Shapiro-Wilk test, non-parametric tests were employed to compare mean ranks among different groups. The Kruskal-Wallis test was used for comparisons involving more than two groups, while the Mann-Whitney U test was used for comparisons between two groups. A p-value of less than 0.05 was considered statistically significant.

## Results

Demographic characteristics

The study included 391 physicians. The majority were aged 25-29 years (51.4%, n=201), followed by those aged 30-39 years (22.8%, n=89). A smaller proportion were aged 40-49 years (17.1%, n=67) and 50-59 years (8.7%, n=34). In terms of gender, 54.5% (n=213) were male and 45.5% (n=178) were female. The nationality distribution showed that 66.8% (n=261) were Saudi and 33.2% (n=130) were non-Saudi. Marital status revealed that 69.8% (n=273) were married, while 30.2% (n=118) were single (Table 1).

**Table 1:** Demographic Characteristics Demographic characteristics of the 391 physicians included in the study.

n=391		N	%
Age (Year)	25 - 29	201	51.4%
	30 - 39	89	22.8%
	40 - 49	67	17.1%
	50 - 59	34	8.7%
Gender	Male	213	54.5%
	Female	178	45.5%
Nationality	Saudi	261	66.8%
	Non-Saudi	130	33.2%
Marital status	Single	118	30.2%
	Married	273	69.8%
Specialty	General practitioner	48	12.3%
	Family medicine	7	1.8%
	Emergency medicine	30	7.7%
	Internal medicine	165	42.2%
	Pediatric medicine	82	21.0%
	Surgery	25	6.4%
	Obstetrics and Gynecology	26	6.6%
	Others	8	2.0%
Place of work	East Jeddah General Hospital	212	54.2%
	King Fahad General Hospital	179	45.8%
Job title	Intern	49	12.5%
	Resident	105	26.9%
	Specialist	189	48.3%
	Consultant	48	12.3%
Smoking		242	61.9%
Physical exercise		235	60.1%

The largest groups were Internal Medicine (42.2%, n=165), Pediatric Medicine (21.0%, n=82), and General Practitioner (12.3%, n=48). Other specialties included Emergency Medicine (7.7%, n=30), Obstetrics and Gynecology (6.6%, n=26), Surgery (6.4%, n=25), and Family Medicine (1.8%, n=7). Place of work was almost evenly split between East Jeddah General Hospital (54.2%, n=212) and King Fahad General Hospital (45.8%, n=179). Job titles were distributed among Interns (12.5%, n=49), Residents (26.9%, n=105), Specialists (48.3%, n=189), and Consultants (12.3%, n=48). Additionally, 61.9% (n=242) of the participants reported smoking, and 60.1% (n=235) engaged in physical exercise (Table 1).

Medical history among participants

Regarding medical history, 56.5% (n=221) had a family history of IBS, 45.0% (n=176) reported that they had been diagnosed with IBS, and 46.3% (n=181) had missed work due to IBS symptoms. The prevalence of food allergies was 49.6% (n=194), and 46.3% (n=181) were using medication. A history of travelers' diarrhea was reported by 53.7% (n=210), and 47.7% (n=174) indicated that travelers' diarrhea was the trigger for the first attack of IBS. Moreover, 59.8% (n=234) had experienced emotional stress in the previous six months (Table 2).

**Table 2:** Prevalence of Medical History Factors Among Participants Prevalence of medical history factors among the 391 physicians. IBS: Inflammatory Bowel Syndrome

n=391	N	%
Family history of IBS	221	56.5%
Have you been diagnosed with IBS	176	45.0%
Absence from work because of IBS symptoms	181	46.3%
Food allergy	194	49.6%
Using medication	181	46.3%
History of travelers' diarrhea	210	53.7%
Was traveler diarrhea the trigger for the first attack of IBS?	174	47.7%
History of emotional stress in the previous 6 months	234	59.8%

Demographic characteristics and their association with Birmingham scores

The Birmingham score varied significantly with age, gender, place of work, job title, smoking status, and physical exercise. Younger age groups (25-29 years, 51.4%, n=201) had higher mean ranks (212.98) compared to older age groups, with the lowest mean rank observed in the 50-59 years age group (8.7%, n=34) (155.93) (p-value = .009). Males 54.5% (n=213) had a significantly higher mean rank (213.37) compared to females 45.5% (n=178) (175.22) (p-value < .001). Place of work also showed significant differences, with King Fahad General Hospital 45.8% (n=179) having a higher mean rank (209.05) compared to East Jeddah General Hospital 54.2% (n=212) (184.98) (p-value = .035). Job titles showed that Interns 12.5% (n=49) had the highest mean rank (231.96), while Consultants 12.3% (n=48) had the lowest (151.09) (p-value = .005). Smokers 61.9% (n=242) had a significantly lower mean rank (166.33) compared to non-smokers 38.1% (n=149) (214.27) (p-value < .001). Those who did not engage in physical exercise 39.9% (n=156) had a higher mean rank (207.67) compared to those who did 60.1% (n=235) (178.42) (p-value = .012) (Table 3).

**Table 3:** Demographic Characteristics and Their Association with Birmingham Scores Kruskal Wallis Test, Mann-Whitney U Test. *. Association is significant at p-value <.05

n=391		Birmingham		
		N	Mean Rank	p-value
Age (Year)	25 - 29	201	212.98	.009*
	30 - 39	89	189.02	
	40 - 49	67	174.69	
	50 - 59	34	155.93	
Gender	Male	213	213.37	< .001
	Female	178	175.22	
Nationality	Saudi	261	188.93	.078
	Non-Saudi	130	210.20	
Marital status	Single	118	210.11	.103
	Married	273	189.90	
Specialty	General practitioner	48	192.35	.643
	Family medicine	7	216.79	
	Emergency medicine	30	176.60	
	Internal medicine	165	201.73	
	Pediatric medicine	82	191.95	
	Surgery	25	193.86	
	Obstetrics and Gynecology	26	217.27	
	Others	8	133.25	
Place of work	East Jeddah General Hospital	212	184.98	.035*
	King Fahad General Hospital	179	209.05	
Job title	Intern	49	231.96	.005*
	Resident	105	196.77	
	Specialist	189	197.65	
	Consultant	48	151.09	
Smoking	Yes	242	166.33	< .001
	No	149	214.27	
Physical exercise	Yes	235	178.42	.012*
	No	144	207.67	

Absence from work due to IBS symptoms

**Figure 1:**
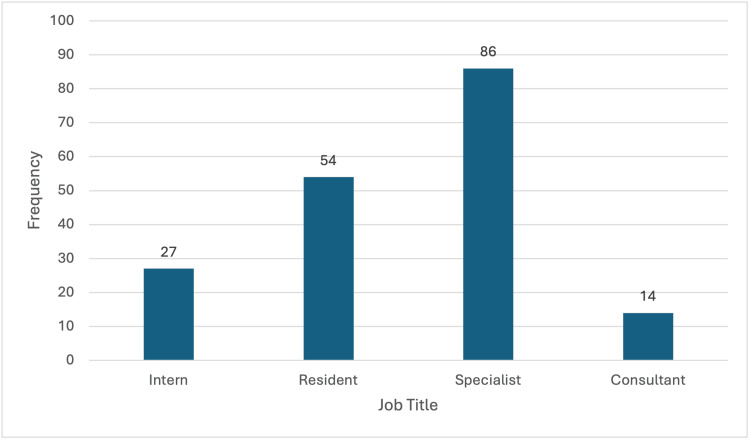
Job Title and Absence from Work Due to IBS Symptoms Distribution of absence from work due to Inflammatory Bowel Syndrome (IBS) symptoms among different physicians’ job titles.

Absence from work due to IBS symptoms was analyzed across job titles. Among interns, 55.1% (27 out of 49) missed work, while 44.9% (22 out of 49) did not. For residents, 51.4% (54 out of 105) missed work, while 48.6% (51 out of 105) did not. Among specialists, 45.5% (86 out of 189) missed work, while 54.5% (103 out of 189) did not. For consultants, 29.2% (14 out of 48) missed work, while 70.8% (34 out of 48) did not (Figure 1).

## Discussion

The findings of the present study showed that the prevalence of IBS was 45% among the included participants. Furthermore, 46.3% of the participants had missed work due to IBS symptoms. This is considerably higher than the global prevalence of IBS which is estimated to be around 11% in the general population [11]. This is also higher compared to the prevalence reported by physicians in Saudi Arabia. For example, a study focusing on board-certified physicians in Saudi Arabia reported a prevalence of 16.3%, with significant associations found between IBS and factors such as long working hours and gender [12]. The study included 594 participants. Similarly, another study by Wells and Chande reported that the prevalence of IBS in resident physicians was 19% which is still quite lower than the present study. They further identified that every one hour of sleep deprivation increases the likelihood of IBS by 1.32 times [13]. 

In the present study, younger age was associated with a significantly increased risk of IBS. This aligns with the previous literature which has shown that the prevalence of IBS decreases with age. The reported prevalence was highest in individuals between the ages of 18-39 years and lowest in individuals above the age of 65 years [14]. IBS affects individuals of all ages including children. In approximately 50% of the individuals diagnosed with IBS, symptoms appear before the age of 35 years and the prevalence is 25% less in individuals who are aged above 50 years compared to younger individuals [15]. 

Gender differences were also significant in the present study, with male physicians having a higher prevalence of IBS symptoms than female physicians. This finding contrasts with much of the existing literature, which generally reports a higher prevalence of IBS among women in the general population. Epidemiological data suggests that the women-to-men ratio for IBS diagnosis ranges from 1.8 to 2: 1, which suggests a significantly higher prevalence in women [16]. Similarly, AlAmeel et al. in their study also reported a higher prevalence of IBS among female participants [12]. The findings of the present study can be explained by the fact that the majority of the participants in the present study were males. Ibrahim et al., however, did not find a significant difference in IBS prevalence based on gender [9]. 

Non-smokers had a significantly higher mean rank for IBS symptoms compared to smokers, which is somewhat counterintuitive given that smoking is generally considered a risk factor for gastrointestinal disorders. Mahmood et al. reported that smoking is a significant risk factor for IBS [17]. Similarly, another study reported that smoking was reported by 36.4% of individuals who had IBS compared to 20.5% who did not which suggests that smoking can cause IBS [18]. However, previous studies that have investigated the prevalence of IBS in health professionals in Saudi Arabia did not find a significant difference based on smoking history [9,12]. 

In the present study, physical exercise was associated with a lower prevalence of IBS symptoms. This aligns with the existing literature as physical activity can decrease the risk of IBS. For example, a study reported that individuals who are physically active are 1.27 times less likely to suffer from IBS compared to those who have a sedentary lifestyle [19]. The current study also analyzed the impact of IBS on work attendance. The findings showed that nearly half (46.3%) of the participants had missed work due to IBS symptoms. This aligns with previous literature which has suggested that work impairment increases with the increase in symptoms of IBS. Furthermore, they identified that anxiety associated with IBS was linked with absenteeism and fatigue in affected individuals [20]. Similarly, another study reported that IBS was associated with greater absenteeism, presenteeism, overall work productivity loss, and activity impairment [21].

Strengths

This study has several strengths that enhance the validity and reliability of its findings. Firstly, the use of a simple random sampling method ensures the sample is representative of the population, reducing selection bias. Secondly, the sample size exceeded the minimum required, increasing the study's statistical power. Thirdly, the inclusion of a comprehensive and validated questionnaire, specifically the Birmingham IBS Symptoms Questionnaire, allows for a thorough assessment of IBS symptoms and their impact. Additionally, the study's focus on a specific and well-defined population-physicians working in general governmental hospitals-provides valuable insights into the prevalence and factors associated with IBS in this group.

Recommendations

Based on the findings of this study, several recommendations can address the high prevalence of IBS among physicians. Firstly, there should be increased awareness and education about IBS and its impact on quality of life and job performance. Hospitals and healthcare institutions should implement regular screening programs to identify and support physicians suffering from IBS.

Additionally, stress management and mental health support should be available to help mitigate the psychological factors that may worsen IBS symptoms. Promoting a healthy lifestyle, including regular physical exercise and smoking cessation, can also be beneficial. Finally, further research is needed to explore the underlying causes of IBS among physicians and develop targeted interventions to reduce its prevalence.

Limitations

This study has several limitations to consider when interpreting the results. Firstly, the cross-sectional design limits the ability to establish causality between IBS and the associated factors. Secondly, the data was self-reported, which may introduce bias due to underreporting or overreporting of symptoms and behaviors. The study was also conducted in a specific geographical area and among a particular group of healthcare professionals, which may limit the generalizability of the findings to other regions and populations. Additionally, the study did not account for all potential confounding factors, such as dietary habits and specific stressors related to the medical profession. Future studies should consider these factors to provide a more comprehensive understanding of IBS prevalence and its determinants among physicians.

## Conclusions

This study highlights the high prevalence of IBS among physicians in Jeddah, Saudi Arabia, with significant associations found between IBS symptoms and various demographic and medical history factors. Younger age, male gender, non-smoking status, and lack of physical exercise were associated with higher IBS prevalence. The findings emphasize the need for increased awareness, regular screening, and targeted interventions to support physicians suffering from IBS. Further research is needed to explore the underlying causes and develop effective strategies to mitigate the impact of IBS on healthcare professionals.
